# Insecticidal and Antifungal Activities of Chemically-Characterized Essential Oils from the Leaves of *Withania frutescens* L.

**DOI:** 10.3390/life12010088

**Published:** 2022-01-08

**Authors:** El Moussaoui Abdelfattah, Allali Aimad, Mohammed Bourhia, Khalid Chebbac, Ahmad Mohammad Salamatullah, Walid Soufan, Hiba-Allah Nafidi, Mourad A. M. Aboul-Soud, Lahcen Ouahmane, Amina Bari

**Affiliations:** 1 Laboratory of Biotechnology, Environment, Agrifood and Health, Faculty of Science, University of Sidi Mohamed Ben Abdellah, Fez 30050, Morocco; abdelfattah.elmoussaoui@usmba.ac.ma (E.M.A.); amna.bari@usmba.ac.ma (A.B.); 2 Laboratory of Animal and Plant Production, and Agro-Industry, Faculty of Sciences, Ibn Tofail University, BP 133, Kenitra 14000, Morocco; aimad.allali@uit.ac.ma; 3 Laboratory of Microbial Biotechnology, Agro-Sciences and Environment (BioMAgE), Cadi Ayyad University, Marrakesh 40000, Morocco; l.ouahmane@uca.ac.ma; 4 Laboratory of Biotechnology Conservation and Valorisation of Natural Resources, Faculty of Sciences Dhar ElMahraz, Sidi Mohammed Ben Abdallah University, Fez 30000, Morocco; khalid.chebbac@usmba.ac.ma; 5 Department of Food Science & Nutrition, College of Food and Agricultural Sciences, King Saud University, 11 P.O. Box 2460, Riyadh 11451, Saudi Arabia; asalamh@ksu.edu.sa; 6 Plant Production Department, College of Food and Agriculture Sciences, King Saud University, P.O. Box 2460, Riyadh 11451, Saudi Arabia; wsoufan@ksu.edu.sa; 7 Department of Food Science, Faculty of Agricultural and Food Sciences, 2325 University Street, Quebec, QC G1V 0A6, Canada; Hiba-allah.nafidi.1@ulaval.ca; 8 Department of Clinical Laboratory Sciences, College of Applied Medical Sciences, King Saud University, P.O. Box 10219, Riyadh 11433, Saudi Arabia; maboulsoud@ksu.edu.sa

**Keywords:** antifungal, insecticidal, *Withania frutescens* L., fungi, *Fusarium oxysporum* f. sp. Ciceris, *Callosobruchus maculatus*

## Abstract

The current study was conducted to investigate antifungal and insecticidal activities of essential oil extracted from the Moroccan *Withania frutescens* L. (EOW), and their chemical composition was profiled. To achieve this goal, EOW was extracted by the hydro-distillation method and their phytochemical constituents were characterized by gas chromatography-mass spectrometry analyses (GC-MS). Insecticidal activity was evaluated by use of four tests: contact toxicity, inhalation toxicity, and repellent tests. Antifungal activity was evaluated on *Fusarium oxysporum* f. sp. Ciceris *(F. oxysporum)* using different concentrations of EOW. GC/MS analysis revealed that EOW was rich in carvacrol (31.87%), thymol (30.08%), and camphor (9.13%). At a 1-µL/L dose, EOW exhibited mortality rates of 23.13 ± 1.07% and 24.41 ± 1.21% against *Callosobruchus maculatus (C.maculatus)* by inhalation and contact, respectively. Notably, EOW dose of 20 μL/L caused significant mortality rates of 95.1 ± 3.5% and 76.69 ± 1.71% by inhalation and contact, respectively. EOW exhibited an inhibitory effect on mycelial growth against the tested fungi *F. oxysporum* of 100% and 93.5 ± 1.1% for the 9 and 4.5 mg/mL doses, respectively. The reduced mycelial growth rate for *F. oxysporum* was recorded to be 0.3 ± 0.1 and 0.6 ± 0.1 mm/h for the EOW doses of 2.25 and 4.5 mg/mL, respectively. The outcome of the present work showed that EOW has a promising antifungal and insecticidal activity, and it can therefore be employed as a natural alternative insecticidal and mycocidal agent to replace the chemically-synthesized ones.

## 1. Introduction

Despite being small, pulse crops represent significant members of the legume family, which includes more than 1800 species. Peas, beans, lentils, chickpeas, and faba beans are examples of pulses, the seeds of which are primarily consumed as food. Pulses are unsurpassed nutritionally-rich vegetable foods in terms of dietary protein content and contain amino acids essential for human nutrition. It is thus fitting that pulses can replace the dietary protein deficiency of animal origin. Some pulses represent a natural source of micronutrients such as vitamins (A, B, C, etc.), trace elements, and mineral salts indispensable for sustaining healthy body functions [1]. However, pulse crops are very often characterized by low and unstable yields during the harvest periods because of their sensitivity to abiotic constraints (cold, heat, soil degradation), biotic constraints (diseases and insect pests), and the rarity of resistant varieties to such constraints [2].

Yield instability is mostly recorded in crops due to the implication of different environmental factors such as abiotic and biotic constraints. In addition, pulses along with legumes may be subject to damage as a result of poor storage conditions. Fungi, molds, and insect pests make post-harvest storage difficult and cause yield loss through a direct decrease in weight and nutritional qualities of the product [3,4]. Post-harvest losses caused by insect pests during storage have been reported to be a growing problem in Africa, leading to the loss of more than 30% of stored dry food products [5].

*Callosobruchus maculatus* (*C. maculatus*) belongs to the cosmopolitan insect pests attacking crops of leguminous plants [6]. The existence of this insect in leguminous stocks may cause a great loss because of its rapid multiplication in warehouses. In this sense, it was reported that *C. maculatus* can affect more than 50% of stock during a few months along with damage to the organoleptic characteristics of products [7,8].

*Fusarium oxysporum (F. oxysporum)* is the most destructive disease of the chickpea crop with huge annual yield losses [9]. For instance, the loses due to this fungal strain reached 12–15% in Spain [10], 40% in Tunisia [11], and 10–50% in Pakistan [12]. *Fusarium* can damage the crop when conditions are favorable for its development [11].

Faced with the threat posed by these insects and fungi, their control is essentially based on the use of chemical insecticides, especially fumigants whose repercussions can be harmful to human health and the biological balance of the ecosystem. The use of chemical insecticides is associated with some serious shortcomings as it is known to cause prominent hazardous non-target toxicity, environmental pollution and can lead to the development resistant strains or superbugs. The search for natural, eco-friendly, and innovative pest-control strategies is justified [13].

In this context, the use of medicinal plants with an insecticidal effect is becoming increasingly important throughout the world due to their eco-friendly charecteristics. Plants are exploited in several forms to limit post-harvest losses either as a crude plant or through its derivatives such as powders, essential oils, plant oils, or even purified compounds. *W. frutescens* L. is an annual woody medicinal plant that belong to Magnoliophyta division, class Magnoliopsida, order Solanales, family Solanaceae, and genera Withania. This genus is indigenous to North Africa, South Asia, Western Asia, Southern Europe, the Mediterranean, and the Canary Islands [14]. Chemical analysis of crude extracts prepared from *W. frutescens* revealed the presence of several potentially bioactive phytoconstituents including polyphenols, tannins, mucilage, terpenoids, flavonoids, and saponins. Previous ethnopharmacological surveys reported the use of *W. frutescens* in controlling disease, namely: tuberculosis, neurodegenerative, conjunctivitis, and cancer [15,16]. Similarity, scientific reports showed that *W. frutescens* possesses several useful medicinalproperties including antifungal, antibacterial, anti-inflammatory, antidiabetic, and wound healing [17]. However, reports on the medicinal properties of EOW are lacking.The present study was therefore initiated to investigate the chemical composition, antifungal, and bio-insecticidal effects of EOW against *F. oxysporum* and adult *C. maculatus*.

## 2. Material and Methods

### 2.1. Plant Material

*W. frutescens* was harvested at the end of March 2019 from the surrounding area of Fez-Morocco. The botanical identification was carried out by Amina Bari (Sidi Mohamed Ben Abdellah University, Morocco). Next, plant specimens were deposited at the herbarium of the University under the voucher number BPRN-69WF. Leaves of *W. frutescens* were dried in the shade for 10 days before being ground into powder prior to extartcion.

#### 2.1.1. Extraction of EOW

**Figure 1:**
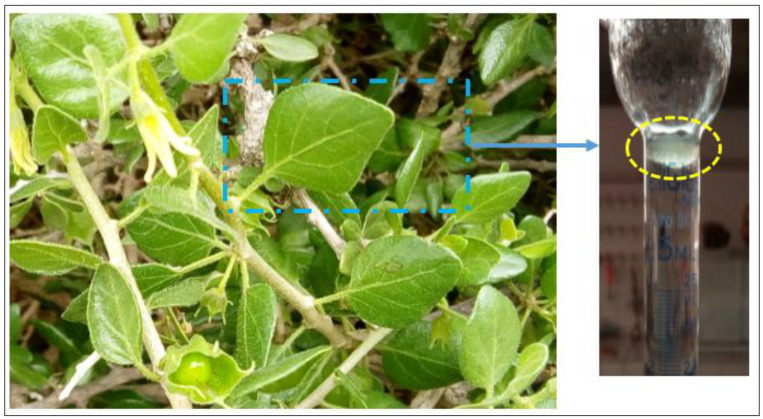
Leaves of *Withania frutescens* L. used for essential oil extraction.

In the present work, the essential oil was obtained from leaves of *W. frutescens* (Figure 1) by the hydro-distillation method using a Clevenger apparatus for 2 h. EOW was dried with anhydrous sodium before being filtered and stored in darkness at 4 °C until further use. The yield of EOW was calculated using the following Formula (1):

YHE = MHE/MD × 100(1)

where YHE is the yield of essential oil (%), MHE is the mass of the EO (g), and MD is the mass of dry plant matter (g)

#### 2.1.2. Gas Chromatography—Flame Ionization Detector (GC-FID)

The extracted oil was diluted with hexane (1:10 dilution) before using 1 µL for chromatographic characterization. A trace gas ghromatograph (GC) equipped with a with HP-5MS non-polar fused silica capillary column (60 m, 0.32 mm, film thickness 0.25 µm) was used to fulfill analysis. The system was programmed with an oven temperature of 50 °C/2 min to 280 C at 5 °C/min, whereas the final temperature was held for 10 min. The injection was done with split mode, ratios 1:20; nitrogen (N_2_) carrier gas, flow rate 1 mL/min. Injector temperatures were set to 250 and 280 °C for the detector (flame ionization detector, FID) [18].

#### 2.1.3. Analysis of the Chemical Composition of EOW by GC/MS

The phytochemical identification of different chemical compounds contained in EOW was carried out by GC-MS. In this sense, the essential oils were analyzed using a Thermo Fischer capillary model coupled with the mass spectrometer system. A non-polar HP-5MS capillary fused silica column with 60 m, 0.32 mm, 0.25 µm film thickness was used to achieve analysis. The operating condition of the GC-MS analysis included an initial temperature of 40 °C/2 min, and a speed of 2 °C/min with a final temperature of 260 °C/10 min; meanwhile, the injector temperature was 250 °C. Further, helium was used as a carrier gas with 1 mL/min. EOW was diluted in hexane with a dilution ratio (10:100). Next, 1 µL was injected with fractional injection and ionization mode. The sweep mass range was m/z 40–650, the ion source temperature 200 °C, ionization energy 70 eV, and interface line temperature 300 °C. The characterization of chemicals was done by determining their retention indices (RI) referring to those of a serial n-alkane counterpart (C8-C20) [19].

### 2.2. Insecticidal Activity of EOW

#### 2.2.1. Breeding of insects

*C. maculatus* used for EOW insecticidal activity testing was acclimatized under the following laboratory conditions: 25 ± 1 °C, 65 ± 5% relative humidity, and a photoperiod of 10:14 h (light/dark). All experiments were performed under the same conditions. Chickpea (*Cicer arietinum*) indigenous to Morocco lands was used for testing.

#### 2.2.2. Toxicity of EOW against Callosobruchus Maculatus

##### Toxicity by Contact

In the present work, 100 g of chickpeas were used for testing purposes. To achieve this goal, grains were infested by five pairs of insects (male and female) of 0–48 h packed in plastic containers (1 μL) tightly closed. Next, EOW was added to the sample at concentrations ranging from 1 to 20 μL/100 g before being shacked for two minutes. Untreated grains confined with *C. maculatus* were used as control. After 48 h of treatment, *C. maculatus* mortality was assessed. Eggs deposited in the grains were counted at 12 days post-treatment, whereas emerged insects were counted regularly at 28 days. The observed mortality rate was corrected by Abbott’s formula [20,21]:

(2) $$\mathrm{Pc}=100\times \frac{\mathrm{Po}-\mathrm{Pt}}{100-\mathrm{Pt}}$$

where Pc is the percentage corrected mortality, Po is the observed mortality in the trial and Pt is the observed mortality in the control.

The percentage decrease in the number of eggs and adults emerged in each concentration of EOW was calculated using the following formula:

(3) $$\mathrm{PR}=\frac{\mathrm{NC}-\mathrm{NT}}{\mathrm{CN}}\times 100$$

where PR is the percent oviposition or decrease in emerged insects, NC is the number of eggs or insects hatched in the control, and NT is the number of eggs or insects hatched in the treatment.

##### Toxicity by Inhalation

In glass jars of 1-L volume, small masses of the cotton were suspended with a thread attached to the inside of the lid. EOW doses of 1, 5, 10, and 20 μL were dropped into the cotton using a micropipette. Next, ten *C. maculatus* (male and female) whose ages ranged from 0 to 48 h were placed into jars with different concertation of oil. For each dose, three replicates were performed. The comparison was made with a control sample (cotton without test solutions). The observed mortality rate was corrected by the formula [21]:

(4) $$\mathrm{Pc}=100\times \frac{P0-\mathrm{Pt}}{100-\mathrm{Pt}}$$

where Pc is the percentage corrected mortality, Po is the observed mortality in the trial, and Pt is the observed mortality in the control.

##### Repulsion Test

The repellent effect of EOW on *C. maculatus* adults was evaluated using the preferential area method on filter paper. Briefly, 9-cm diameter discs prepared from filter paper were used for this purpose. Discs were uniformly immersed with 0.5 mL of EOW at different doses (1, 5, 10, and 20 µL/mL) corresponding to 0.016; 0.079; 0.157; and 0.315 µL/disc per disc. Discs immersed with a similar volume of acetone were used for control. Next, the Petri dishes were closed with Parafilm before being incubated for 30 min. The number of bruchids present on the half of the disc treated with EOW was counted against the number on the untreated part. Three replicates for each experiment were conducted under the same environmental conditions as the insect rearing.

The percentage of repulsion (PR) was calculated according to the following formula [22]:

(5) $$\mathrm{PR}=\frac{\mathrm{NC}-\mathrm{NT}}{\mathrm{NC}+\mathrm{NT}}\times 100$$

where PR is the percent repellency (%), NC is the number of insects in the control area and NT is the number of insects in the treatment area.

### 2.3. Antifungal Activity of EOW

In the present work, the direct contact method was used to investigate the antifungal effect of the essential oil extracted from the aerial part (leaves) of *W. frutescens*.

#### 2.3.1. Preparation of the Suspension Cultures

In the present work, *Fusarium oxysporum* (BMFS19) was kindly provided by the Lab of Biotechnology (Faculty of Science, Fez, Morocco). Next, spores of 5 days cultures were seeded on the Petri dishes including one milliliter of sterile physiological water. Next, the obtained suspension was estimated by measuring the optical density (OD) using a spectrophotometer at 630 nm before being adjusted to obtain a suspension with 10^7^ spores/mL [23].

#### 2.3.2. Preparation of Culture Media and Incubation of Petri Dishes

Media were prepared by mixing different concentrations (*v*/*v*) of EOW and 0.2% agar and potato dextrose agar (PDA) medium (Table 1). The obtained solution was autoclaved at 120 °C for 20 min [24,25]. Next, 10 µL of spore inoculum were seeded onto the Petri dishes except for the control medium. Thereafter, the Petri dishes were sealed with parafilm before being incubated for 6 days at a temperature of about 26.5°C [23,24].

#### 2.3.3. Evaluation of Mycelial Growth

The evolution of the mycelial growth of *Fusarium oxysporum* was carried out each day by measuring the diameter of the mycelial colony taking into consideration the controlled growth. In this sense, the growth inhibition rate (IY) was calculated according to the following formula [23,24].

(6) $$\mathrm{IY}=\frac{\mathrm{Dt}-\mathrm{Ds}}{\mathrm{Dt}}\times 100$$

where Dt is the diameter of colonies without EO and Ds is the diameter of colonies after being treated with EO.

##### Determination of Minimum Inhibitory Concentrations

Determination of minimum inhibitory concentrations (MIC) of EOW against the studied fungal was effectuated according to the previously reported protocols [26,27]. Briefly, Petri dishes with concentrations that showed a complete absence of mycelial growth were selected to determine the minimum inhibitory concentrations (MICs).

##### Determination of the Mycelial Growth Rate (MG)

The mycelial growth rate of each concentration was determined by the formula [28]:

(7) Cs=(D1/T1)+(D2−D1)/T2)+⋯+(Dn−D(n−1)/Tn)

where Dn is the diameter of growth measured each day in millimeters and Tn is the time expressed in days.

### 2.4. Statistical Analysis

The statistical ananlysis was conducted with one-way ANOVA and the reults obtained are expressed as means plus standard deviations. In addition, Tukey’s test was used as post hoc test for multiple comparions. Significant values were considered when P was less than 0.05.

## 3. Results and Discussion

### 3.1. Identification of EOW Composition by GC/MS

**Figure 2:**
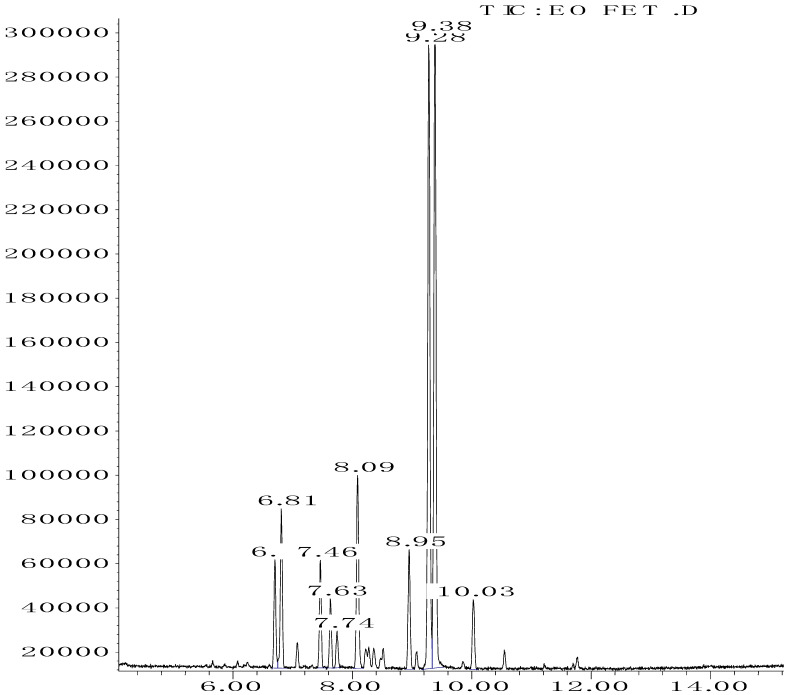
Chromatography profile of EOW identified by GCMS.

The phytochemicals identified in EOW by GC/MS are summarized in Figure 2 and Table 2. The extraction yield of EOW was about 0.28%, which is reasonable relative to species among *Solanaceas* [18]. This yield is comparable to some plants that are industrially-exploited as a source of essential oils such as *Latin rosa* L.(0.1–0.35%), *Salvia rosmarinus* L. (1–2.5%), *Mentha piperita* L. (0.5–1%), *Citrus Aurantium* L. (0.5–1%), *Lavandula angustifolia* L. (0.8–2.8%), *Pimpinella anisum* L. (1–3%), and *Thymus vulgaris* L.(2–2.75%) [29]. In addition, the chemical composition of EOW contained certain chemicals that were also reported in similar studies on the chemical composition of plants; such as pulegone compounds [30,31,32].

The difference in the extraction yield between plants can be ascribed for the implication of different environmental factors [33]. The findings of the chemical analysis revealed that the studied EOW was notably composed of many potentially active phytochemicals such as Carvacrol (31.87%), Thymol (30.076%), and Camphor (9.13%). Taken together, our results were found to be consistent with previous reports [18], which demonstrated the presence of Camphor, Thymol, and Carvacrol as major chemical consituents of EOW [18].

### 3.2. Insecticidal Activity

**Figure 3:**
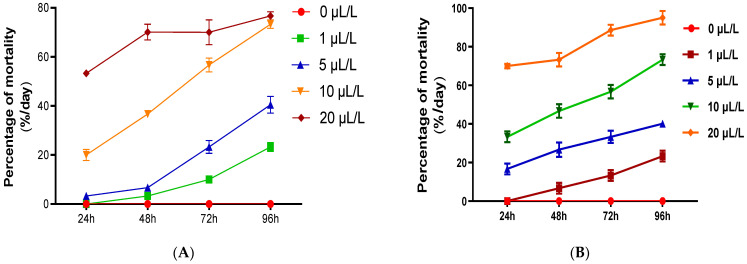
Mean percentage mortality of *C. maculatus* adults exposed to increasing doses (µL/L) of essential oils extracted from *Withania* (EOW) by inhalation (**A**) and by contact (**B**) as a function of increasing duration (h) of exposure (insects-EOW).

In the present work, different dosages of EOW (0.1, 5, 10, and 20 μL/L air volume) were used to determine its toxicity against *C. maculatus* by use of ihalation test, as previosuly described in erlier work [34]. Adult mortality of *C. maculatus* was recorded every 24 h for four days, and the findings are given in Figure 3.

Results revealed that the lowest concentration (1 μL/L air volume.) of EOW resulted in 23.13 ± 1.07% and 24.41 ± 1.21% mortality in adult *C.maculatus* by inhalation and contact, respectively, 96 h post-exposure (Figure 3). The highest concentration (20 μL/L air volume) caused mortality rate of 95.1 ± 3.5% and 76.69 ± 1.71% by inhalation and contact, respectively. By contrast, no insect mortalities were observed in the control. LC_50_ value obtained for inhalation test was determined to be 13.28 ± 2.62 μL/L air volume, which is lower than that obtained with contact test (18.41 ± 1.29 μL/L).

**Figure 4:**
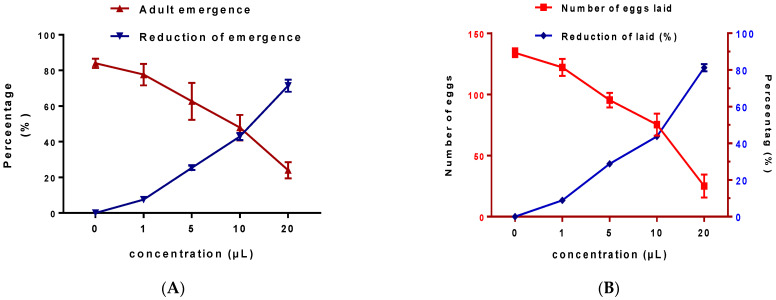
Effects EOW on *C. maculatus* emergence. *C. maculatus* exposed to EOW by inhalation (**A**) and by contact (**B**).

In the bioassay, egg laying of *C. maculatus* occurred within 24 hrs of mating and the number of eggs laid varied according to applied doses of EOW. A significant dose-dependent reduction in egg-laying behavior, where a maximum reduction of 81.26 ± 2.01%, was recorded with the 20 µL EOW/L air volume (Figure 4B). It was evident that EOW caused significant reduction in the viability rate of eggs relative to the control, reaching 84.0 ± 2.65% with the maximum dose of 20 µL EOW/L (Figure 4A). Taken together, our results were found to be consistent with those reported elsewhere [34]; whereby essential oil isolated from *Mentha pulegium* L. resuted in adverse effects on the egg-laying behavior of *C. maculatus.*

**Figure 5:**
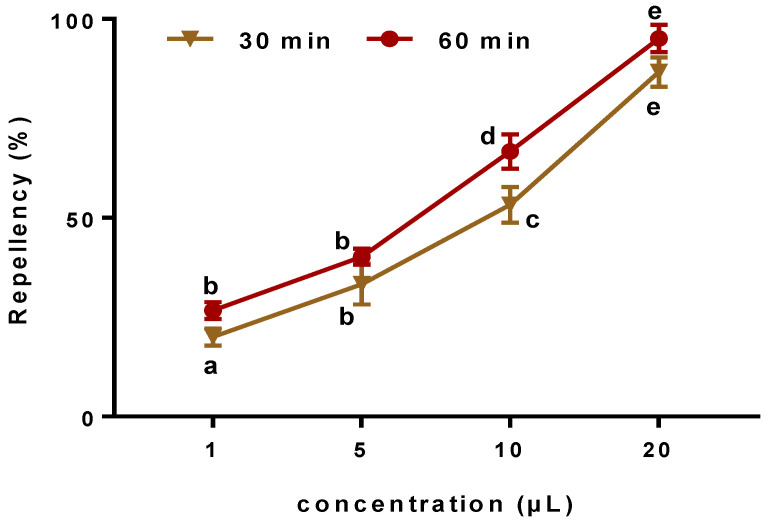
Repellent activity of different concentrations of *EOW* against *C. maculates.* Class a (0.1 ≤ repellency ≤ 20%); Class b (20.1 ≤ repellency ≤ 40%); Class c (40.1 ≤ repellency ≤ 60%; Class d (60.1 ≤ repellency ≤ 80%; Class e (80.1 ≤ repellency ≤ 100%.

The results represented here showed that the EOW under investigation exhibited strongly repellent activity against *C. maculatus* with a rate of 95.12 ± 3.42%, in dose-dependent manner (Figure 5). Therefore, the potential of EOW as a valuable source of ecofriendly insecticidal agents against *C. maculatus* has been confirmed. It is evident that pest control strategies relying upon the use of chemically-synthesized products have been associated with alarmingly growing environmental concerns. Therefore, it is primitive to switch to pest-control products of natural origin such as plant-derived essential oils; which could be a game-changer in fighting pests due to their powerful insecticidal activityagainst different larval stages [35]. One major advantage of using natural essential oils extracted from plants is their wide safety margins as they are characterized as being environmentally friendly insecticides and are considered a valuable source for the development of novel natural bioactive agents as an effective alternative to synthetic ones [36].

Several studies have documented the beneficial action of essential oils on reducing longevity of pest species in stored grains including *C. maculatus*. Because of their high volatility, essential oils along with their constituents, particularly monoterpenes, exert insecticidal effects and disrupt insect growth at different life stages [28,29,30]. Efficacy of essential oils varies according to their phytochemical profiles and the target insect, e.g., bean sprout, is more sensitive to phenolic monoterpenes [36,37,38,39]. Indeed, monoterpene mixtures are neurotoxic agents acting on different targets. For example, it has been reported that slinalool caused a reduction in the amplitude and frequency of the action potential, whereby leads to a reduction in the post-hyperpolarization phase that follows the transmission of the nerve impulse. By contrast, estragol has been reported to exhibit more specific reduction in the post-hyperpolarization phase. The simultaneous action of the two compounds caused paralysis and eventually the death of the insects [39]. On the other hand, the effect of essential oils on reproduction is the result of both an inhibition of oogenesis and an increase in the retention of eggs in females lateral oviducts. In this context, previous studies have reported the modifications in the environment surrounding the oviposition sites can lead to inhibition of oogenesis and retention of eggs in oviducts [32,33].

### 3.3. Antifungal Activity of EOW

**Figure 6:**
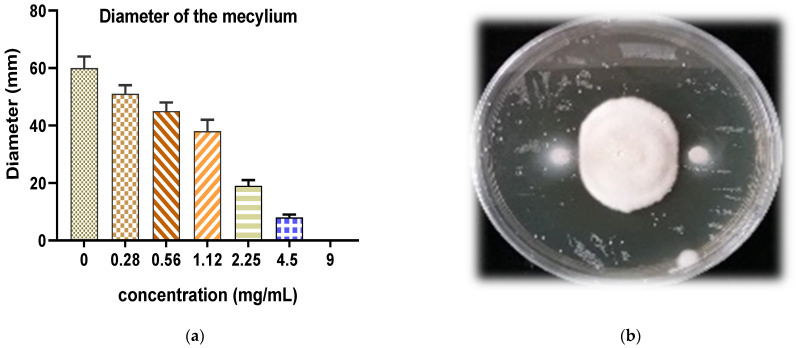

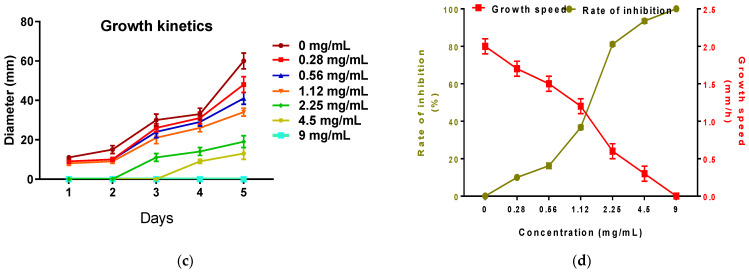
Results of the antifungal activity of *EOW* on *Fusarium oxysporum* f. sp. (**a**) Effect of EOW on *Fusarium oxysporum* f. sp mecylium; (**b**) Phograph of *Fusarium oxysporum* f. sp; (**c**) Effect of EOW on *Fusarium oxysporum* f. sp growth kinetics; (**d**) Effect of EOW on *Fusarium oxysporum* f. sp growth speed and inhibition rate.

The mycelial growth of the studied fungal strain *F. oxysporum* was significant in the absence of EOW, resulting in mycelial diameter of 60 mm (Figure 6a). However, this growth was strongly inhibited in the presence of EOW in a dose-dependent manner (0.28 to 9 mg/mL). Mycelial growth was evaluated as a function of both time and EOW concentration tested by measuring the diameter of fungal mycelium (Figure 6b). Figure 6 clearly shows a dose-dependent significant inhibition of mycelial growth in the presence of EOW compared to the negative control (0 mg/mL). Concerning the kinetics of mycelial growth, it was noticed that mycelial growth differed according to the concertation applied, such that the lowest EOW concentrations (2.28 and 4.5 mg/mL) permitted mycelial growth, which had gradually started from the second day of incubation. However, total inhibition of mycelial growth was recorded with EOW maximum concentration of 9 mg/mL (Figure 6c). From this figure, it can be seen that the absence of essential oil allowed mycelial growth from the first day of incubation compared to the control (Figure 6c). Therefore, it can be concluded that EOW has a potent fungistatic effect in a dose-dependent manner. Fungal inhibition rates of EOW are represented in Figure 6d, depictingthe evaluation of the inhibition percentage of the studied essential oil. A significant inhibitory activity leading to a maximal fungicidal effect (100% total inhibition) was observed at the EOW concentration of 9 mg/mL (Figure 6d). Hence, it can be concluded that EOW strongly controlled *F. oxysporum* mycelial growth rate exhibiting both fungistatic and fungicidal potencies that were dependent on the concentration used. For example, it was noticed that the inhibition of *F. oxysporum* growth rate increased with increasing oil concentration so that a total growth rate inhibition was recorded for the maximum EOW concentration of 9 mg/mL. Thus, the obtained results unequivocally confirm that the essential oil under investigation exhibits effective growth-control potencies against *F. oxysporum* in a dose-dependent fashion. Based on the investigated parameters in the current study, such as mycelial growth, mycelial growth kinetics, inhibition rates, and mycelial growth rate, we can confirm that EOW has strong antifungal activity against *F. oxysporum*.

This observed strong fungicidal activity exerted by EOW against the studied fungal strain cloud be ascribed for its chemical constituents, particularly thymol, which was detected by GC-MS. These results were corroborated by those reported in earlier studies [18,27], which showed that thymol and carvacrol are among the most active compounds in essential oils against microbes [18,27]. These two molecules have a very broad spectrum of antimicrobial activity and are naturally-present in the essential oils of most plant species [40,41,42,43]. The mechanism-of-action whereby these natural compounds kill fungi has been investigated elsewhere [44,45,46]. Essential oils rich in phenolic compounds have been reported to cause phenol toxicity against fungi is by the inactivation of fungal enzymes that have SH group in their active sites [44,45,46]. Phenolic terpenes are also involved by binding to the amine and hydroxylamine groups of microbial membrane proteins, causing alteration of permeability, and eventually leading to leakage of intracellular constituents [47,48,49,50].

## 4. Conclusions

The use of natural aromatic products derived from medicinal plants as fungal and pest control strategies can have several valuable advantages over current synthetic products. The outcome of the present work suggests that EOW has powerful insecticidal and antifungal activities, which could be explained by its richness in potentially bioactive compounds such as carvacrol and thymol. Further detailed investigations on the ecofriendly essential oil extracted from Moroccan *W. frutescens*, in terms of toxicity against non-target organisms along with testing its purified compounds, are highly warrented.

## Figures and Tables

**Table 1:** Preparation of the culture medium.

Medium	PDA (mL)	EOW (mL)	Agar 0.2% (mL)	Concentration (mL)
Control	40.00	0.0	0.0	0
1	36.00	0.4000	3.6000	1/100
2	38.00	0.2000	1.8000	1/200
3	39.00	0.1000	0.9000	1/400
4	39.50	0.0500	0.4500	1/800
5	39.75	0.0250	0.2250	1/1600
6	39.75	0.0125	0.1125	1/3200

**Table 2:** Phytochemical composition of EOW identified by GCMS.

Peak	R.T (min)	Name	Formula	Area (%)	RI	Chemical Structure
1	6.70	Camphene	C10H16	4.42	946	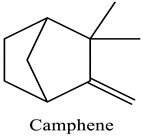
2	6.81	1,8-Cineole	C10H18O	6.93	1031	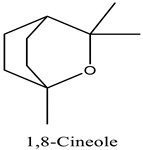
3	7.47	Fenchone	C10H16O	4.43	1086	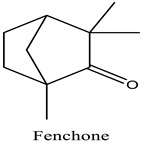
4	7.63	α-Thujone	C10H16O	2.88	1102	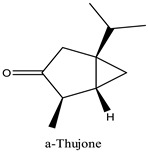
5	7.74	β-Thujone	C10H16O	1.53	1114	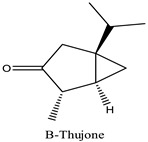
6	8.09	Camphor	C10H16O	9.13	1146	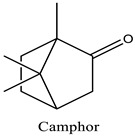
7	8.95	Pulegone	C10H16O	5.37	1237	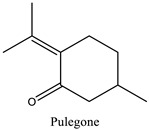
8	9.28	Thymol	C10H14O	30.08	1290	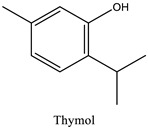
9	9.39	Carvacrol	C10H14O	31.87	1299	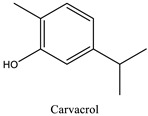
10	10.03	Piperitenone oxide	C10H14O2	3.37	1368	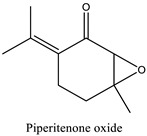

## Data Availability

All data reported here is available from the authors upon request.
